# Evaluation of clinical, laboratory and morphologic prognostic factors in colon cancer

**DOI:** 10.1186/1477-7819-6-98

**Published:** 2008-09-08

**Authors:** Michele Grande, Giovanni Milito, Grazia Maria Attinà, Federica Cadeddu, Marco Gallinella Muzi, Casimiro Nigro, Francesco Rulli, Attilio Maria Farinon

**Affiliations:** 1 University Hospital Tor Vergata, department of surgery, University hospital Tor Vergata, Viale Oxford, 81 00133 Rome, Italy

## Abstract

**Background:**

The long-term prognosis of patients with colon cancer is dependent on many factors. To investigate the influence of a series of clinical, laboratory and morphological variables on prognosis of colon carcinoma we conducted a retrospective analysis of our data.

**Methods:**

Ninety-two patients with colon cancer, who underwent surgical resection between January 1999 and December 2001, were analyzed. On survival analysis, demographics, clinical, laboratory and pathomorphological parameters were tested for their potential prognostic value. Furthermore, univariate and multivariate analysis of the above mentioned data were performed considering the depth of tumour invasion into the bowel wall as independent variable.

**Results:**

On survival analysis we found that depth of tumour invasion (P < 0.001; F-ratio 2.11), type of operation (P < 0.001; F-ratio 3.51) and CT scanning (P < 0.001; F-ratio 5.21) were predictors of survival. Considering the degree of mural invasion as independent variable, on univariate analysis, we observed that mucorrhea, anismus, hematocrit, WBC count, fibrinogen value and CT scanning were significantly related to the degree of mural invasion of the cancer. On the multivariate analysis, fibrinogen value was the most statistically significant variable (P < 0.001) with the highest F-ratio (F-ratio 5.86). Finally, in the present study, the tumour site was significantly related neither to the survival nor to the mural invasion of the tumour.

**Conclusion:**

The various clinical, laboratory and patho-morphological parameters showed different prognostic value for colon carcinoma. In the future, preoperative prognostic markers will probably gain relevance in order to make a proper choice between surgery, chemotherapy and radiotherapy. Nevertheless, current data do not provide sufficient evidence for preoperative stratification of high and low risk patients. Further assessments in prospective large studies are warranted.

## Introduction

Advances in the management of colon cancer over the past decades have resulted in an improvement of the prognosis of the disease. The proportion of stage I and II has increased from 39.6% to 56.6% leading to a raise of five-year relative survival from 33% in 1970s to 55.3% in 1990s [1].

Nevertheless, the five-year survival rate of colon cancer has not improved dramatically in the last decade, remaining at approximately 60%, and colon cancer is still one of the leading killers in the Western countries [2].

In truth, despite curative resection, many patients develop recurrence at the primary site or distant organs. These high risk patients could be candidates for more aggressive treatments (neoadjuvant chemotherapy) in order to improve the prognosis [3]. This target requires not only the development of new therapeutic modalities but also a reliable preoperative stratification of high and low risk patients.

Prognostic factors derived from clinical, laboratory and pathologic data of colorectal cancer patients have been considered important and have been investigated in order to make a proper choice between surgery chemotherapy and radiotherapy, but the results of the previous studies were often intriguing and conflicting [4,5].

Actually, most studies investigating prognostic factors for large bowel cancers did not distinguish between the subpopulation of colon and rectal cancer, despite the different biological characteristics, treatment modalities, pattern of recurrence and survival rates of the two group of neoplasms [6].

Further, it was suggested that proximal and distal colon cancer can differ in histopathologic characteristics, molecular pattern, stage of diagnosis and, consequently, clinical outcome. Over the past 20 years, the literature has demonstrated a stage migration of colorectal cancer from distal to proximal sites with a tendency for proximal tumours to present at a more advanced stage than distal tumours [7].

At the moment, the most accurate prognostic factor remains the extension of the tumour into the bowel wall as expressed in the Dukes classification or TNM classification [2,4].

The main endpoint of the present study was to evaluate the prognostic implication of many preoperative clinical, laboratory and patho-morphological data by both univariate and multivariate analysis.

Therefore, we performed two statistical analysis of clinical, laboratory and patho-morphological data in a group of patients with colon cancer, considering survival and pT staging as the independent variables.

## Materials and methods

### Patients

A total of 103 patients with colon cancer, who were surgically treated between January 1999 and December 2001 at the Department of General Surgery, University Hospital Tor Vergata, Rome, were evaluated for eligibility.

Patients who suffered from rectal cancer, colon carcinoma with locally advanced invasion (pT4) or colon cancer with distant metastasis were excluded. Only elective surgery cases were considered. Thus, out of 103 subjects, 92 patients, who underwent to curative resection and were followed for at least 5 years, were analysed.

Preoperative staging was performed using colonoscopy, conventional transabdominal ultrasonography, CT scan of abdomen, barium enema, chest X-ray and blood tests that included tumour markers.

CT scanning was performed using oral and intravenous contrast. Patients were scanned at 5-mm intervals from the diaphragm through the pubic symphysis. We did not use a three-dimensional endoluminal view; we used only transverse CT images. The assessment of extracolonic compartment metastases of the abdomen and pelvis was performed on 5-mm venous phase contrast-enhanced transverse images.

Bowel wall thickening of more than 0.5 cm was considered to indicate the presence of a neoplasm. Colorectal wall invasion was analyzed according to a modified T classification reported by Filippone et al. Contrast-enhanced CT criteria for T staging were ≤*T2 *= smooth outer border of thickened colorectal wall with a clear surrounding fat plane, *T3 *= tumor with rounded or nodular advancing margin, *T4 *= obliteration of fat planes between colorectal tumor and adjacent organs. This classification was used to address known limitations at CT in distinguishing T1 and T2 lesions.

The cancer was found in the ileocecal junction of 11 patients (11.9%), in ascending colon of 14 patients (15.2%), in transverse colon of 8 subjects (8.7%), in hepatic flexure of 8 patients (8.7%), in splenic flexure of 7 patients (7.6%), in descending colon of 10 patients (10.9%) and in sigmoid colon of 34 patients (36,9%).

### Operative procedure

All patients were surgically treated. Right hemicolectomy was performed in 14 patients, ileocecal resection in 11 cases, transverse colon resection in 8 patients, left hemicolectomy in 25 patients, sigmoid resection in 24 patients, Hartmann procedure in 10 cases.

T and N staging was based on the international TNM classification, as follows: pT1, tumor invading submucosal layer; pT2, tumor invading muscularis propria or subserosa; pT3, tumor penetrating serosa and perivisceral fat; and pT4, tumor invading adjacent organs. Lymph nodes were likewise classified: N0, no regional lymph node metastasis; N1, metastasis in one to three perirectal lymph nodes; N2, metastasis in four or more perirectal lymph nodes; and N3, metastasis in pelvic lymph nodes. The patients were classified as follows: 5 pT1 N0 M0; 2 pT1 N1 M0; 4 pT1 N2 M0; 16 pT2 N0 M0;; 2 pT2 N1 M0; 2 pT2 N2 M0; 37 pT3 N0 M0; 14 pT3 N1 M0; 10 pT3 N2 M0.

### Data analysis

Medical records of patients with colon cancer were isolated in a computerized database. The database included 53 demographics, clinical, laboratory and patho-morphological parameters: name, sex, age, symptoms and major medical problems of patients; laboratory data and neoplastic markers values; location, size, endoscopic appearance and preoperative staging of the tumour; operation type, degree of differentiation and pTNM of the cancer; postoperative course, recurrence, and condition at follow-up.

These patients returned for follow-up every 6 months during the first 3 years and then once a year. When necessary, telephone contact was made with the patient to obtain up-to-date information. The dead line of follow-up was up to January 2007. The longest follow-up time was 96 months with an average period of 60 months.

Statistical analysis was performed using the software program Statgraphics Plus. Analysis of variance (F-ratio) was used for comparison of quantitative parameters, whereas qualitative parameters were analyzed by Chi-square test. To compare the prognostic value of the statistically significant variables, multivariate analysis (Multivariate analysis of variance MANOVA) was performed with 95% CI for the means of each variable.

All *P *values were two-tailed. *P *values of less than 0.05 were considered statistically significant.

## Results

Between January 1999 and December 2001, 92 patients with colon cancer underwent surgical resection at the Department of General Surgery, University Hospital Tor Vergata, Rome and were included in the study.

Patients consisted of 48 males and 44 females who ranged in age from 37 to 94 years (average age of 69.2 years). The average interval between symptoms and diagnosis was 5.4 months (range 1–70).

In our experience 92 patients with colon cancer were recruited between January 1999 and December 2001 and followed up to December 2006. The average follow up period of the 92 patients was 40.6 months (range 3–96). The 5-year survival rate was 39.1% (36/92).

The results of univariate analyses of clinical, laboratory and pathomorphologic data considering pT staging as the independent variable are summarized in table 1; pT stages were considered as 2 categories: pT < 2 and pT > 2.

**Table 1:** Results of univariate analyses of clinical, laboratory and pathomorphologic data considering pT staging as independent parameter

**Source**	**Sum of squares**	**Df**	**Mean Square**	**R-ratio**	**P-value**
**pT staging vs mucorrea**					
Between groups	41.2011	1	41.2011	8.75	0.005
Within groups	423.875	90	4.70972		
**Total correct**	465.076	91			

**pT staging vs anismus x**					
Between groups	21.0117	1	21.0117	4.26	0.0519
Within groups	444,064	90	4,93405		
**Within groups**	465,076	91			

**pT staging vs-fibrinogens**					
Between groups	453.687	78	5.8165	6.64	0.0003
Within groups	11.3889	13	0.876068		
**Total (correct)**	465.076	91			

**pT staging vs WBC**					
Between groups	630.923	50	12.6185	1.90	0.01
Within groups	279.55	42	6.65595		
**Total (correct)**	910.473	92			

**pT staging vs hematocrit**					
Between groups	852.117	79	10.7863	2.54	0.05
Within groups	59.5	14	4.25		
**Total (correct)**	911.617	93			

**pT vs CT staging**					
Between groups	126.287	5	25.2575	5.21	0.01
Within groups	58.1571	12	4.84643		
**Total (correct)**	184.444	17			

Presence of mucorrhea and anismus, hematocrit value ranging between 16,7% and 31%, WBC count between 4500 and 5800/mm^3 ^and fibrinogen value > 400 mg/dl were significantly related to pT staging > 2. Further the degree of CT scan T-staging were significantly related to pT staging of the tumour.

Among clinical parameters, presence of mucorrhea (p < 0.005; F-ratio 8.75) appeared more significantly related to pT staging > 2 than anismus (p < 0.05; F-ratio 4.26). Among laboratory data, fibrinogen value > 400 mg/dl (p < 0.0005; F-ratio 6,64) appeared more significantly related to pT > 2 than WBC count ranging between 4500 and 5800/mm^3 ^(p < 0.01; F-ratio 1,90) or hematocrit value of 16,7–31% (p < 0.05; F-ratio 2,54). Furthermore, CT scan-T staging appeared strongly related to the pathologic-T staging (p < 0.01; F-ratio 5,21).

Only those variables that appeared significant in the univariate analysis were considered for the multivariable analysis. Fibrinogen value appeared the most significant predictor of pathologic-T staging of the tumour (p < 0.001, F-ratio 5.86), as shown in table 2.

**Table 2:** Results of multivariate analysis of clinical and laboratory data of patients with colon cancer

**Source**	**Sum of squares**	**Df**	**Mean Square**	**F-ratio**	**P-value**
Main effects					
A: fibrinogens	405.233	78	5.19529	5.86	0.0014
B: mucorrhea	1.38889	1	1.38889	1.57	0.2366
C: tenesmus	0.25	1	0.25	0.28	0.6059

**Residual**	9.75	11	0.886364		

**Total (correct)**	465.076	91			

On survival analyses, the pathologic-T staging of the tumour (p < 0.01; F-ratio 2.11), the operation type (p < 0.01; F-ratio 3.51) and the CT scanning (p < 0.05; F-ratio 5,21) appeared to be prognostic indicators (table 3).

**Table 3:** Prognostic indicators of survival in patients surgically treated for colon cancer

**Source**	**Sum of squares**	**Df**	**Mean Square**	**R-ratio**	**P-value**
**Survival vs pT staging**					
Between groups	136.587	15	9.10583	2.11	0.01
Within groups	328.489	76	4.32222		
**Total correct**	465.076	91			

**Survival vs operation**					
Between groups	22292.1	6	3715.35	3.51	0.01
Within groups	89913.6	85	1057.81		
**Total correct**	112206.0	91			

**Survival vs CT staging**					
Between groups	7770.25	5	1554.05	3.90	0.05
Within groups	4778.86	12	398.238		
**Total correct**	12549.1	17			

Finally, tumour site was found significantly related neither to survival not to the degree of tumour differentiation as shown in table 4.

**Table 4:** Statistical correlation between tumour site and degree of differentiation on the cancer

**Degree of differentiation**	**Site**		**Total**	**Significance**
	**R**	**L**		
**very differentiated**	**5**	**10**	**15**	**p = n.s.**
**moderately differentiated**	**27**	**42**	**69**	**p = n.s.**
**insufficient differentiated**	**5**	**3**	**8**	**p = n.s.**

**Total**	**37**	**55**	**92**	

## Discussion

Several studies provided data regarding the survival of patients with colorectal cancer. Different clinico-pathological prognostic factors have been proposed: age, location of the cancer, surgical procedure, radical resection, blood transfusion, pathological type, diameter, depth of tumor invasion, lymph node metastasis and distant metastasis [1,2,8].

The site of the tumor is one of the prognostic factors investigated. Patients with colon cancer are considered having a better survival than those with rectal cancer [6,7,9]. In previous studies distal location and advanced stage of tumor were determined as independent prognostic factors for survival of patients with colorectal cancer [10]. In the present study, we considered only patients with colon cancer and, among these patients, we found relationship neither between tumor location and survival nor between tumor location and degree of cancer differentiation.

Differently, pathological classification is one of the prognostic factors proposed for patients with colorectal cancer. It was suggested that patients with different papillary adenocarcinoma have the best outcome, patients with moderately-differentiated and mucinous adenocarcinoma have a moderate outcome, patients with signet-ring cell poorly-differentiated adenocarcinoma have a poor prognosis [11].

Current data indicates also that radical resection and type of operation are important prognostic factors. In a recent study on prognosis of 96 patients with colon cancer, survival time and 3-year survival rate were, respectively, 24 months and 6.5% for patients who received R2 procedure, 98 months and 87.9% for patients who underwent R0 resection [1]. Further, the prognosis of the patients undergone left hemicolectomy (splenic flexure of colon, descending colon and most part of sigma colon) was not different from that of the patients undergone right hemicolectomy (caecum, ascending colon and hepatic flexure of colon)[1]. Differently, in our experience type of operation was an indicator of survival (P < 0.001; F-ratio 3.51).

Several analyses confirmed the vital importance of tumour stage, as reflected in Dukes or TNM classification, in predicting survival [2,4,5]. The overall 5-year survival rate of patients with colorectal cancer, reported in literature, is at least 60% and raises to 90% for Dukes A tumour and conversely decreases to 10% for Dukes D^2^.

Accordingly, stage T4 and vascular invasion were reported as markers of poor prognosis [12]. Petersen and colleagues [13] identified these factors in a series of 268 patients with stage II colonic cancer, together with positive surgical margins and perforation. Burdy et al [14] identified stage T4 in a study of 108 colonic cancers, and also reported independent significance for male sex, bowel obstruction and number of nodes examined. Morris et al [12], in a observational study on 1306 patients, reported only T4 and vascular invasion as significant factors in multivariable analysis. Mulcahy et al [15] found a trend for prognostic significance of vascular invasion in rectal but not in colonic cancers. Accordingly, in our experience the pT staging of the cancer was strongly related to survival (P < 0.001; F-ratio 2.11). Therefore, histopathological factors continue to be the most valuable source of information regarding the possible evolution of patients with colon cancer [16,17].

Although surgical therapy is the basis of treatment for patients with colon cancer, multimodal therapeutical concepts are currently applied not only in metastatic disease and Dukes C patients but also in Dukes B [18-20]. Further, the introduction of new drugs has extended the therapeutic options [21,22] and ongoing studies with novel targeted therapies will show their results in the next years.

Other open questions include the timing of treatment (neoadjuvant treatment for locally advanced cancer), the duration of therapy and whether there is a real possibility of stratifying patients for neoadjuvant treatment on the basis of preoperative prognostic factors [23-25].

Actually, recent studies, that underlined remission rates as high as 40% in advanced colon cancer and the efficacy of adjuvant chemotherapy, are now leading to revaluation of the therapeutic approach to colon cancer. Data from animal models and tumour biologic hypotheses also point to a possible advantage for preoperative therapy to improve disease-free survival and overall survival in colon cancer patients [26].

Therefore, the main target of this investigation was to identify preoperative clinical, laboratory and patho-morphological parameters that may be indicators of the pT staging of the tumour.

Considering clinical data, we observed that the presence of mucorrhea and anismus were indicators of pT staging > 2, it was suggested that these signs are also related to the tumour size. On laboratory, we found that hematocrit value between 16,7 and 31% and WBC count ranging between 4500 and 5800/mm^3 ^were significantly associated to pT > 2. Further fibrinogen value > 400 mg/dl both on univariate and multivariate analysis was significantly associated to pT staging > 2; however the importance of fibrinogen value to predict tumour invasion and prognosis of these patients remains uncertain, given that this marker is not specific and it is also involved in acute-phase reaction.

The significance of increase of CEA and CA 19-9 levels to predict the prognosis of the patients remains a problem for debate; in our experience CEA and CA 19-9 levels were found indicators neither of pT staging nor of survival. Differently, in a recent study on 103 patients with colorectal carcinoma, Nozoe et al [27] observed that high preoperative CEA and CA 19-9 were predictors of survival. On the opposite, in a large study of 279 patients with colon cancer and 293 patients with rectal cancer, Tominaga et al [8] found that, although higher preoperative CEA level group tended to have a higher recurrence rate, preoperative CEA level was not statistically related to the prognosis of both groups of patients (Hazard ratio 1.34 and 1.35).

Besides this, an interesting result of our investigation is the importance of CT scan preoperative staging both as predictors of survival and of pT staging. These data confirm the diagnostic accuracy of this tool not only for the rectum but also for the colon cancer as pointed out by Blomqvist in a recent review on advances in preoperative staging of colorectal cancer patients. The author underlines the technological advancement of CT scan related to hardware, software, development of CT colonography, new contrast enhancement agents [28,29].

In summary, these data confirm the vital importance of tumour stage in predicting survival and recurrence and provide the grounds for further work in order to assess the prognostic significance of various clinical, laboratory, patho-morphological markers and to define the subgroups of patients at different risk of recurrence who could be treated more or less intensively.

## Competing interests

The authors declare that they have no competing interests.

## Authors' contributions

MG: manuscript preparation and critical review. GM: critical review. GMG: data collection and manuscript preparation. FC: literature review and manuscript preparation. MGM: literature review. CN: data collection and literature review. FR: critical review. AMF: critical review. All authors read and approved the final manuscript.
